# Income-, education- and gender-related inequalities in out-of-pocket health-care payments for 65+ patients - a systematic review

**DOI:** 10.1186/1475-9276-9-20

**Published:** 2010-08-11

**Authors:** Sandro Corrieri, Dirk Heider, Herbert Matschinger, Thomas Lehnert, Elke Raum, Hans-Helmut König

**Affiliations:** 1 University of Leipzig, Health Economics Research Unit, Department of Psychiatry, Liebigstr. 26, 04103 Leipzig, Germany; 2 Department of Medical Sociology and Health Economics, University Medical Center Hamburg-Eppendorf, Martinistr. 52, 20246 Hamburg, Germany; 3 University of Leipzig, Department of Psychiatry, Semmelweisstr. 10, 04103 Leipzig, Germany; 4 Division of Clinical Epidemiology and Aging Research, German Cancer Research Center, Bergheimer Str. 20, 69115 Heidelberg, Germany

## Abstract

**Background:**

In all OECD countries, there is a trend to increasing patients' copayments in order to balance rising overall health-care costs. This systematic review focuses on inequalities concerning the amount of out-of-pocket payments (OOPP) associated with income, education or gender in the Elderly aged 65+.

**Methods:**

Based on an online search (PubMed), 29 studies providing information on OOPP of 65+ beneficiaries in relation to income, education and gender were reviewed.

**Results:**

Low-income individuals pay the highest OOPP in relation to their earnings. Prescription drugs account for the biggest share. A lower educational level is associated with higher OOPP for prescription drugs and a higher probability of insufficient insurance protection. Generally, women face higher OOPP due to their lower income and lower labour participation rate, as well as less employer-sponsored health-care.

**Conclusions:**

While most studies found educational and gender inequalities to be associated with income, there might also be effects induced solely by education; for example, an unhealthy lifestyle leading to higher payments for lower-educated people, or exclusively gender-induced effects, like sex-specific illnesses. Based on the considered studies, an explanation for inequalities in OOPP by these factors remains ambiguous.

## Background

In all OECD countries, there is a trend to increasing patients' copayments in order to balance rising overall health-care costs [1]. Major concerns in this topic revolve around inequalities in burden for subgroups of society, being unproportionally charged for health care services because of their socioeconomic background. The difference in financial strain is displayed in a larger share of income that must be invested in health care services, leading to dissimilar efforts for comparable benefits, and disadvantages for low-income beneficiaries.

There are three major forms of copayments. Firstly, there is a varying amount that must be paid by the patient before the insurance company steps in, called deductible. Regularly, a higher deductible is associated with a lower premium, leaving the beneficiary with a lower basic amount, but at higher risk in case of morbidity. Secondly, the co-insurance marks the amount of OOPP the beneficiary has to spend after the deductible limit is reached. The insurer only pays a stipulated percentage share of the costs, while the patient pays for the rest. Thirdly, and in the focus of this article, there are direct OOPP for health-care services. Examples are costs for prescription medications, hospital stays, alternative medicine, physiotherapy or home nursing, which are not covered by insurance policies and have to be paid by the patients themselves [2]. All three forms of copayments are suspected to evoke or reinforce inequalities in burdens for beneficiaries, especially regarding predispositions in education, sex and, foremost, income, as will be explored in this review. In the USA, copayments have been established for a long time and have caused a large body of studies, making the USA the most valuable source for literature. This may give the opportunity to outline possible future developments in Europe. The present review gives an overview of the inequalities of OOPP by the fastest growing population, the elderly aged 65+, associated with income, education and sex. In the elderly, inequalities are likely to be most apparent due to extensive use of medical services caused by age-related morbidity. Purpose of this task is to provide a basis, serving as foundation for future studies focusing on the mechanisms causing the described inequalities.

## Methods

### Search strategy

**Figure 1:**
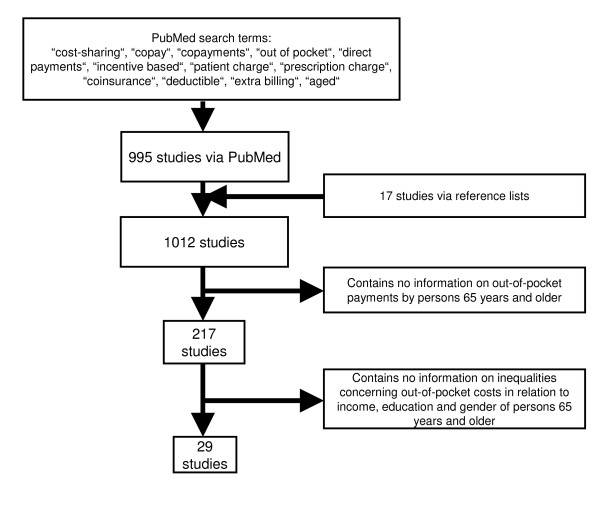
**diagram showing filtering process of literature used**.

As shown in Figure 1, an online PubMed search was conducted to identify studies. Search terms included combinations of the following keywords: "cost sharing"[All Fields] OR "copay"[All Fields] OR "copayments"[All Fields] OR "out of pocket"[All Fields] OR "direct payments"[All Fields] OR "incentive based"[All Fields] OR "patient charge"[All Fields] OR "prescription charge"[All Fields] OR "coinsurance"[All Fields] OR "deductible"[All Fields] OR "extra billing"[All Fields] AND "aged"[MeSH Terms]. The search process ended on November 9^th^, 2009. Studies' abstracts were examined in detail, and, if required, extended by a full text revision.

### Inclusion and exclusion criteria

Unless separate data analyses were conducted for the 65+ subsamples, studies not limited to the senior population (defined as 65 years of age or older) were excluded. Only studies in English and German language based on evaluation of primary data were considered. Finally, articles had to contain relevant information concerning education, sex and/or income in relation to out-of-pocket payments for people 65+ (as illustrated in Figure 1).

### Analysis of data

To facilitate comparisons, all cost estimates were inflated to 2008 US dollars using the Chain Type Price Index for Gross Domestic Product (US Census Bureau 2010). The following information was systematically extracted (see Table 1): authors, year of publication, sample size indicating the validity of the article (varying from 113 to 30,791,751 persons), study design, analyzed outcome, key findings stratified according to income, education and gender, and confounders controlled for.

**Table 1:** Overview of studies reviewed (in alphabetical order)

Study	Sample	Study Design	Outcomes
Adams, Soumerai, & Ross-Degnan, 2001a	4439 Medicare beneficiaries with hypertension	national longitudinal survey (MCBS 1995); inferential statistic	Association between types of drug coverage, consumption & costs per tablet; Findings: income
Blustein, 1995a	4110 female Medicare beneficiaries	probability survey based on multistage, stratified cluster sample of Medicare (MCBS 1991-1992); multiple inferential statistic	Use of mammography during first 2 years of Medicare offered benefit; Findings: income, education
Blustein, 2000b	4334 Medicare beneficiaries with hypertension	nationally-representative face-to-face survey of Medicare; multiple inferential statistic	Sexual differences in burden for prescription drugs; Findings: sex
Chandra et al., 2007	70912 CalPers plan members	Panel of Medicare supplemental plan Members (CalPers 2000-2003); multiple inferential statistic	Influence & consequences of price elasticity in patient cost-sharing; Findings: income
Crystal, Johnson, Harman, Sambamoorthi, & Kumar, 2000b	7886 Medicare beneficiaries	nationally representative survey of Medicare, stratified, multistage, area probability sample (MCBS 1995); multiple inferential statistic	Overview on size, distribution & burden of OOPP; Findings: income, education
Davis, Poisal, Chulis, Zarabozo, & Cooper, 1999a	12.000 Medicare beneficiaries	Panel Survey (MCBS 1995); descriptive	Overview on sources & extent of drug coverage among Medicare beneficiaries; Findings: income
Dowd et al., 1994a	2891 Medicare HMO & fee-for-service members	Survey; multiple inferential statistic	Characteristics of Medicare beneficiaries & influence on choice of health plan; Findings: income
Fahlman, Lynn, Doberman, Gabel, & Finch, 2006d	4602 Medicare beneficiaries	Cross-sectional, retrospective review & 1990 Census data; multiple inferential statistic	Drug spending by disease & demographics in last year of life; Findings: income
Gellad, Huskamp, Phillips, & Haas, 2006a	5596 Medicare beneficiaries	Panel Survey, nationally representative sample (MEPS-HC 1996-2000); multiple inferential statistic	Estimation of change of OOPP for drugs after Part D implementation; Findings: income
Goldman & Zissimopoulos, 2003b	7836 Medicare beneficiaries	Cross sectional survey of 4th wave of Panel survey (HRS 1998); inferential statistic	Examination of OOPP health-care spending; Findings: income
Guidry, Aday, Zhang, & Winn, 1998b	593 Texan cancer patients	analytical cross-sectional survey; inferential statistic	Prevalence of barriers to cancer treatment; Findings: income
Hwang, Weller, Ireys, & Anderson, 2001a	22.326 patients with chronic condition	cross-sectional survey (MEPS 1996); descriptive	Impact of chronic condition & demographics on OOPP spending; Findings: sex
Klein, Turvey, & Wallace, 2004i	6535 participants of AHEAD-study	cross-sectional study of 2nd wave of AHEAD study 1997; inferential statistic	Reasons for delay in medication use because of cost; Findings: income, sex
Lapsley, March, Tribe, Cross, & Brooks, 2001a	113 patients with osteo-arthritis in Australia	prospective-cohort study; inferential statistic	OOPP expenditures related to osteo-arthritis; Findings: sex
McGarry & Schoeni, 2005b	3821 >70 years old Americans (271 widowers, 3550 married)	national panel survey (2 Waves) (HRS); descriptive	Financial gap between widowed and married Elders; Findings: sex
Miller & Champion, 1993a	161 women	convenience sample, mailed survey; inferential statistic	Relationship of patient's characteristics and mammography utilization; Findings: income, education
Mitchell, Mathews, Hunt, Cobb, & Watson, 2001a	499 patients with at least one regular prescription medication	cross-sectional survey; mutliple inferential statistic	extent of mismanaging of prescription drugs among rural Elders; Findings: income
Mojtabai & Olfson, 2003c	10.413 Medicare beneficiaries	cross-sectional (HRS 2000); multiple inferential statistic	Association between drug coverage & adherence; cost-related poor adherence & health outcomes; Findings: income
Ness, Cirillo, Weir, Nisly, & Wallace, 2005b	1099 participants of HRS study	cross-sectional (HRS 2000); inferential statistic	Correlates of complementary & alternative medicine (CAM) utilization among Elders; Findings: sex
Pourat, Rice, Kominski, & Snyder, 2000d	15.103 Medicare beneficiaries	cross-sectional (MCBS 1996); inferential statistic	Comparison of supplemental insurances to examine impact of socioeconomics; Findings: income, education
Rector & Venus, 2004a	1500 Medicare+Choice plan beneficiaries	cross-sectional, random sample in eight Medicare+Choice Plans; inferential statistic	Influence of drug benefits on affordability for beneficiaries; Findings: income
Rice & Desmond, 2006e	9278 Medicare beneficiaries	cross-sectional (SIPP 2001); descriptive	Number and characteristics of Medicare beneficiaries excluded from low-income subsidies because of failed asset test; Findings: education, sex
Riley, 2008b	4000 Medicare beneficiaries at a time	panel 4 waves (MCBS 1992, 96, 2000, 04); inferential statistic	Trends in OOPP health-care costs for MediCare beneficiaries; Findings: income
Rogowski, Lillard, & Kington, 1997b	996 Elders	cross-sectional (PSID 1990); multiple inferential statistic	Amount & influence of supplemental insurance on burden of prescription drug OOPP costs; Findings: income, education, sex
Sambamoorthi, Shea, & Crystal, 2003b	8814 Medicare beneficiaries	cross-sectional (MCBS 1997); multiple inferential statistic	Total and OOPP burden for prescription drugs in relation to characteristics of elderly population; Findings: income, education
Saver, Doescher, Jackson, & Fishman, 2004d	4492 Medicare+Choice enrollees	cross-sectional survey and administrative data from Medicare, 2000; multiple inferential statistic	Relationship between drug benefit status & access to medications + influence of income; Findings: income, education
Selden & Banthin, 2003b	5733 (1987), 2549 (1996) >65 years old beneficiaries	stratified random samples (NMES 1987 and MPES 1996), longitudinal; descriptive	Amount health-care burden for Elders; Findings: income, sex
Soumerai et al., 2006a	13.835 Medicare beneficiaries	stratified, multistage sample (MCBS 2004), cross-sectional; multiple inferential statistic	Prevalence of cost-related medication non-adherence prior to Medicare Part D; Findings: income
Wei, Akincigil, Crystal, & Sambamoorthi, 2006a	76.440 person-years (30.375 beneficiaries) of Medicare beneficiaries	longitudinal (MCBS 1992-2000); multiple inferential statistic	Gender differences in OOPP expenditures & burden for medication; Findings: sex

### Presentation of findings

This review is structured as follows: every socio-demographic variable has its own chapter describing its association with OOPP. As income contains the most information, the respective chapter is further structured by overall expenditures, and their biggest share, prescription drugs. Furthermore, details on impacts like insurance types or cost-reducing strategies are reported. After describing the association of education and sex with OOPP, a conclusion at the end of this review summarizes all findings, confronts them with theories to put them in scientific perspective, and gives implications.

## Results

### Reviewed Articles

995 papers were found via PubMed, and 17 papers were found via bibliographic search in reference lists of eligible articles, resulting in a total of 1012 studies. After exclusion of studies not focusing on out-of-pocket payments for people 65 years of age or older, 217 studies remained. Out of these, 29 articles remained containing relevant information concerning education, sex and/or income in relation to out-of-pocket payments for people 65+ (as illustrated in Figure 1). Included are 11 longitudinal and 18 cross-sectional surveys, of which 9 articles use the MCBS (Medicare Current Beneficiary Survey), making it the most utilized source of data. Five studies are purely descriptive [3-7], while the remaining 24 use inferential statistics to test differences in out-of-pocket payments between income, education and gender groups. Of these, 14 controlled for confounders, meaning independent control variables were evaluated (for respective confounders in articles see columns in Tables 2, 3 and 4). The articles used in this review originate from 1993-2009, from which 21 were published before the implementation of the Medicare Prescription Drug, Improvement, and Modernization Act of 2003, an important break marking the inclusion of a voluntary drug benefit (Part D), taking effect in January 2006 [8]. Studies varied in analysis of absolute expenditures and its relation to income [9-11], or just focusing on the burden [7,12,13].

**Table 2:** Overview of studies concerning INCOME (in alphabetical order)

Study	Key findings	Confounders controlled for
Adams, Soumerai, & Ross-Degnan, 2001a	high income > good insurance > lower OOPP > higher drug consumption	none
Blustein, 1995a	low income > less probability of mammography	age, race, education, self-rated health status, total Medicare Part B reimbursement in 1991, smoking status, living arrangement
Chandra et al., 2007	low income > high price elasticity > increased hospital visits due to less prevention	type of insurance plan, age, spending tercile, Charlson Index, health status
Crystal, Johnson, Harman, Sambamoorthi, & Kumar, 2000b	average OOPP burden: 19% (lowest quintile: 31.5%, top quintile: 8.5%)	sex, race, age, education, marital status, self-reported health status, number of medical conditions, number of ADL & IADL impairments, insurance coverage
Davis, Poisal, Chulis, Zarabozo, & Cooper, 1999a	high income > best insurance > lowest OOPP	none
Dowd et al., 1994a	high income > best insurance > lowest OOPP	age, sex, marital status, education, living arrangements, number & proximity of living children, health insurance, self-reported health condition
Fahlman, Lynn, Doberman, Gabel, & Finch, 2006d	high income > high utilization & OOPP	race, sex, Charlson Index, age, insurance type
Gellad, Huskamp, Phillips, & Haas, 2006a	Medicare Part D > general cost decline, but: high incomes advantaged through lower burden in Donut Hole	race, chronic conditions, insurance coverage
Goldman & Zissimopoulos, 2003b	high income > high absolute OOPP, but lower burden (highest quartile: 1% OOPP of income, lowest: 17% (up to 43%); hardest hit: those shortly above limit of Medicaid support)	none
Guidry, Aday, Zhang, & Winn, 1998b	disadvantages for minorities (lower income, bad insurance, higher costs, less treatments)	none
Klein, Turvey, & Wallace, 2004i	low income > bad insurance > high OOPP > less prevention > more illnesses > more OOPP > more cost-reducing strategies > high follow-up costs (each +100$/month OOPP > +10% of unregular use)	none
Miller & Champion, 1993a	high income > high utilization & drug adherence	none
Mitchell, Mathews, Hunt, Cobb, & Watson, 2001a	less income > less medication adherence due to OOPP > worse health status & less health consciousness > higher OOPP > less adherence	age, race, education, residential status, health status, medication profile
Mojtabai & Olfson, 2003c	lower income > less adherence	age, sex, race, education, marital status, employment, insurance coverage
Pourat, Rice, Kominski, & Snyder, 2000d	low income > less supplemental prescription drug coverage > high OOPP	none
Rector & Venus, 2004a	low income > more cost induced delay or stop of medication utilization (<$1000 monthly household income: 38%, >$4000: 17%)	none
Riley, 2008b	1992-2004: absolute OOPP up by 22.5%; highest burden: second lowest quartile > no Medicaid	none
Rogowski, Lillard, & Kington, 1997b	low income > higher expenditures & higher burden: 5,4-5,9%, middle income: 1.6%, highest income: 0,6%; insurance coverage reduces amount spent by 50%; cost distribution highly skewed: 55% spend 1% or less, 1% spend 25% of yearly income	age, sex, race, education, residential status, marital status, insurance coverage, health status
Sambamoorthi, Shea, & Crystal, 2003b	Absolute OOPP nearly equal, but: low income > higher burden (+10% burden: <200% of poverty level: 13.4%, >200%: 2.4%)	sex, race, age, education, marital status, insurance coverage, self-rated health status, place of residence
Saver, Doescher, Jackson, & Fishman, 2004d	high income > higher probability of drug benefit (25% vs. 17%) > more adherence	age, race, sex, education, household configuration, insurance coverage, self-rated health status
Selden & Banthin, 2003b	lower income > higher burden: +40% burden 1987 (1996) (below poverty line: 20.9% (19.6%), >200% of p.l.: 3.8% (4.8%))	none
Soumerai et al., 2006a	low income > less drug adherence (<$10.000 yearly income: 14.5%, >$40.000: 8.7%)	sex, age, race, education, self-rated health status, insurance coverage

**Table 3:** Overview of studies concerning EDUCATION (in alphabetical order)

Study	Key findings	Confounders controlled for
Blustein, 1995a	low education > less probability of mammography	age, race, income, self-rated health status, total Medicare Part B reimbursement in 1991, smoking status, living arrangement
Crystal, Johnson, Harman, Sambamoorthi, & Kumar, 2000b	OOPP burden with no high school: 21.4%, college degree: 12.8%	gender, race, age, income, marital status, self-reported health status, number of medical conditions, number of ADL & IADL impairments, insurance coverage
Miller & Champion, 1993a	college degree significant for mammography & physician visits > less OOPP burden in the long-term	none
Pourat, Rice, Kominski, & Snyder, 2000d	better education > better insurance > less OOPP	none
Rice & Desmond, 2006e	higher education than lowest income group > income above Medicaid limit > same OOPP as high income group, but less education & income; higher OOPP than subsidy group for having higher education & income	none
Rogowski, Lillard, & Kington, 1997b	better education > less OOPP burden (higher income, better insurance): >12 years: 1.6%, <12 years: 4.5%	age, sex, race, income, residential status, marital status, insurance coverage, health status
Sambamoorthi, Shea, & Crystal, 2003b	less education > higher OOPP (over 10% of burden without high school degree: 12.1%, college: 3.9%)	gender, race, age, income, marital status, insurance coverage, self-rated health status, place of residence
Saver, Doescher, Jackson, & Fishman, 2004d)	better education > more prescription drug coverage > less OOPP	age, race, sex, income, household configuration, insurance coverage, self-rated health status

**Table 4:** Overview of studies concerning SEX (in alphabetical order)

Study	Key findings	Confounders controlled for
Blustein, 2000b	women > rather poor (26% below poverty line, men: 11%); less employed > less insurance coverage > higher OOPP (18% higher than men for drugs)	age, race, education, self-rated health status, insurance coverage
Fahlman, Lynn, Doberman, Gabel, & Finch, 2006d	women > higher OOPP in last year of life ($668 vs. $586)	race, income, Charlson Index, age, insurance type
Hwang, Weller, Ireys, & Anderson, 2001a	women > longer lifespan > higher probability of comorbidities > higher OOPP	none
Klein, Turvey, & Wallace, 2004i	women > higher OOPP > more cost-reducing strategies	none
Lapsley, March, Tribe, Cross, & Brooks, 2001a	women > higher OOPP for drugs & devices	none
McGarry & Schoeni, 2005b	women > longer lifespan > more widowhood; lowest income quartile (<$12.000): 70% of income spent in final two years for health-care (average: 30%); poverty rate: widows 17%, married Elders: 5%	none
Ness, Cirillo, Weir, Nisly, & Wallace, 2005b	women > more CAM utilization > higher OOPP	none
Rice & Desmond, 2006e	women > longer lifespan: partner dies > income plummets >heir above limit > no subsidies > old-age poverty; of 46% widowers failing asset test > 46% female	none
Rogowski, Lillard, & Kington, 1997b	women > equal expenditures, but higher burden (3.3% vs. 2.8%)	age, income, race, education, residential status, marital status, insurance coverage, health status
Sambamoorthi, Shea, & Crystal, 2003b	women > higher OOPP (over 10% of burden > women 9.4%, men 5.7%)	income, race, age, education, marital status, insurance coverage, self-rated health status, place of residence
Selden & Banthin, 2003b	women > higher burden (over 20% of burden 1987 (1996): 19.6% (19.8%), men: 12.7% (15.9%)	none
Wei, Akincigil, Crystal, & Sambamoorthi, 2006a	women > lower income, more utilization, higher absolute OOPP, higher burden; gender-specific illnesses > less generous benefits > higher OOPP	race, age, marital status, education, place of residence, poverty status, insurance coverage, health status

### Income

#### Overall expenditures

Concerning absolute expenditures, Goldman *et al*. [9] found that mean yearly OOPP in 1998 were slightly higher for people with high income (above $49250/year) than for those with low income (less than $15969/year): $2821 against $2346. A similar situation can be observed for high wealth (above $405.554 assets) compared to low wealth beneficiaries (less than $52.595): $2857 against $2551. Considering OOPP in relation to income, Crystal *et al*. [12] found that in 1995 low-income beneficiaries had lower absolute payment amounts, but faced a significantly higher burden. The lowest income quintile is most affected by high OOPP: in contrast to the top quintile, they spent 31.5% of their yearly income ($1639) compared to 8.5% ($3219) in the top quintile. Selden *et al*. [7] confirmed this finding for 1996: while 19.6% of families living below the poverty line faced expenditures of at least 40% of their household income, only 4.8% of those owning 200% of the poverty line and more belong to this group. When wealth is included as a variable increasing income and spending possibilities of the 65+ population, Goldman *et al*. [9] showed that in 1998 the lowest quartile spent on average 17% of their annual wealth, while the top quartile only less than 1%. 10% of the bottom quartile even spent 43% or more in two years. The development over time supports this argument. Riley [13] found an overall OOPP increase of 0.7% from 1992 to 2004, which was skewed in regard to income quartiles: while the highest quartile experienced a rise of 0.8%, the second lowest quartile was hardest affected by an increase of 2.3%. The lowest quartile is protected by supportive Medicaid coverage.

#### Prescription Drugs

Regarding absolute expenditures, Sambamoorthi *et al*. [10] found only slight differences in mean OOPP for different income groups in 1997: while those living above 200% of the poverty line faced payments of $447 for their prescription drugs, those below had to pay $442. Rogowski *et al*. [11] calculated out-of-pocket drug expenditures at $390 for high-, $492 for middle-, and $850 for low-income beneficiaries. Concerning the burden of prescription drugs use, Rogowski *et al*. [11] found that the overall distribution of OOPP was highly skewed in 1997: 55% of beneficiaries spent 1% or less, while 1% of patients spent more than 25% of their yearly household income on prescription drugs. The same study also shows that the burden of prescription drug costs for high-income beneficiaries was around 0.6%, and 1.6% or even 5.9% for middle and low incomes respectively, resulting in a ten times difference between income groups. According to Sambamoorthi *et al*. [10], nearly 8% of beneficiaries spent more than 10% of their income on prescription drugs only. While 2.4% of those living above 200% of the poverty level were concerned, 13.4% of beneficiaries living below this mark were affected.

#### Insurance type

In this context, a decisive argument is the type of insurance that can be afforded, identifying income as highest influence on choice, and therefore finally on OOPP. Following the 1995 MCBS (Medicare Current Beneficiary Survey), approximately 65% of Medicare beneficiaries had supplemental, prescription drug covering insurance [3]. As Dowd *et al*. [14] point out: those with the lowest income are most likely the ones not able to afford adequate insurance. Widespread basic Medicare coverage is the cheapest way to be insured (low premiums), as long as there are no health expenditures (high deductibles and co-insurance rates), which is most likely to happen to exactly these subgroups. Pourat *et al*. [15] show that with rising income, the proportion of beneficiaries with supplemental insurance rises. Among people relying on basic Medicare fee-for-service, only 5% have yearly incomes above $32.561, but 17% have incomes under $13.024. Adams *et al*. [16] found that state- and employer-sponsored drug coverage, and therefore lower OOPP, lead to a higher consumption of clinically essential drugs. Confirming this finding, Fahlman *et al*. [17] found that increasing levels of household income correlated with a 21% increase of prescriptions and a 25% increase in mean OOPP. The implementation of Medicare Part D in 2006 led to comparable absolute savings for all beneficiaries. Gellad *et al*. [18] found the overall out-of-pocket costs to decline $237 on average, even $501 for seniors without employer-sponsored drug coverage. But in relation to income, the Donut Hole is disadvantaging those 3.4 million low-income seniors who reached it in 2007, having the same costs as high-income beneficiaries, but higher burdens. Considering this, Medicare Part D is not able to reduce barriers to adequate medication use.

#### Cost-reducing strategies

Hence, strategies to reduce costs are prevalent in low-income layers of society. Many Elders relying on their prescription drugs take less medication than prescribed, or do not fill their prescription to save money. This results in worse health conditions that require further treatments and therefore higher OOPP in the long run. Other strategies are accumulation of debt, utilization of medicine only in emergency cases, or asking practitioners for free samples [19]. This is emphasized by Klein *et al*. [8], who found that low-income Elders only insured by Medicare fee-for-service are much more likely to economize. 23.3% of the examined beneficiaries reduce the amount of medication in order not to exceed their prescription cap, stopping total adherence by as much as 16.3%. $100 higher costs per month lead to a 10% higher probability of irregular continuation. Regarding insurance coverage, Saver *et al*. [20] strongly associated income with having a prescription drug benefit. In 2000, 25% of those without a prescription drug benefit experienced non-adherence, compared to only 17% owning such. Rector *et al*. [21] state that in 2002, 38% of people with a monthly household income below $1000 economized, while only 17% of those living with more than $4000 did. Comparable numbers are reported by Soumerai *et al *[22] shortly before the implementation of Medicare Part D: beneficiaries living with less than $10.000 yearly income showed a non-adherence prevalence of 14.5%, those with incomes above $40.000 report only 8.7%. For Medicare beneficiaries living with out-of-pocket drug spending of at least $1000 in 2000, Mojtabai *et al*. [23] stated that only 6% of those living at least 400% above the poverty line economized, while 21% below poverty line admitted to doing so.

### Education

Several articles refer to education as a significant cause for differences among older persons and their burden of OOPP for medical services. The main finding is that the lower the level of education, the higher the burden becomes [10-12]. Lower education may lead to lower income and lack of employer based health programs [15,20]. Concerning the burden of overall out-of-pocket costs in 1995, Crystal *et al*. [12] found that beneficiaries with college degrees spent 12.8% of their income on health-care, while those without a high school diploma spent 21.4%. Focusing on burden of prescription drugs costs only, Rogowski *et al*. [11] found that beneficiaries with less than 12 years of education paid 4.5% of their incomes on prescription drugs in 1990, while those with more than 12 years of education only 1.6%. As suggested before, this was mainly caused by lower retirement incomes and less prevalence of private insurance. Sambamoorthi *et al*. [10] found that 12.1% of those without a high school diploma had to pay more than 10% of their income on prescription drugs in 1997, but only 3.9% of seniors with college education had to. Conversely, a burden of 0-5% was determined for 56.9% of those without a high school diploma, while 77.8% of college graduates belonged to this group. Increments of education confirm this trend proportionally. Regarding insurance coverage, Pourat *et al*. [15] found in 2000 that the likelihood of an employment-based or MediGap coverage is raised by better education, while a poor education increases the probability of basic Medicare fee-for-service insurance only, or no coverage at all, leading to higher OOPP in case of morbidity. Saver *et al*. [20] confirmed this observation in 2004: only 39% of those without coverage faced monthly expenditures below $50, while 79% of patients with private prescription coverage did. Equivalently, 8% of not-covered beneficiaries faced more than $100 per month, while nobody of the second group had to pay this amount. The poorest are secured by Medicaid, but the ones shortly above the income limit for eligibility are confronted with catastrophic costs that eat up all their savings before Medicaid steps in [9]. Another point is the Medicare Part D asset test, denoting specific income and asset thresholds set to qualify for low-income subsidies [6]. Those low-income seniors failing the test mostly have better education than those who qualify for subsidies (college degree: 9.7% vs. 4.4%). But because approximately 70% of those only have incomes less than 135% of the federal poverty line, and around 50% of them only have assets less than $35.000 above the allowing thresholds. These assets would not pay a single year of nursing home care. Compared to beneficiaries without any assets, they have to deal with higher OOPP without governmental support, and in relation to those with higher incomes and larger assets, they have to pay similar OOPP with less means [6]. Furthermore, a low educational standard is associated with lower utilisation of preventive measures. In various studies [24,25] clarify that a college degree is highly associated with preventive behaviour like mammography. Not using preventive measures finally leads to higher OOPP due to worse health status.

### Sex

There are certain gender related differences concerning OOPP. Selden *et al*. [7] found that in women, prevalence of high burden for medical services is significantly higher than in men for all age groups. For example, 12.2% of females aged 75 years and older faced burdens larger than at least 40% of after-tax disposable income in 1996, but only 9.2% of men. Concerning prescription drugs, in 1995 women had 18% more annual OOPP than men ($551 vs. $454) [26]. In a longitudinal study from 1992-2000, Wei *et al*. [27] found the similar inequality ($526 vs. $432) in expenditures. Regarding the burden of OOPP for prescription drugs, Wei *et al*. describe a gender difference of 28% (4.4% vs. 3.2%) [27]. While finding equal absolute OOPP in 1996, Rogowski *et al*. [11] approve the finding that women have a higher relative burden (3.3% vs. 2.8%). Sambamoorthi *et al*. [10] found that 9.4% of women faced a burden of at least 10% of their income for OOPP on prescription drugs, compared to only 5.7% in men. The more distinct the gender differences in health status and income are, the higher the difference in burden appears. One reason for the differences is that women are less likely to be employed, and thereby generate less wealth. Even if women pursue a career, on average they have significantly lower income than men. Wei *et al*. stated that in 1999 the mean income for women aged at least 65 years was $19.097, compared to $35.676 for men [27]. 26% of female Medicare beneficiaries live below the federal poverty line, whereas only 11% of men. Being less often employed also means fewer possibilities to be covered by an employer-financed supplemental insurance (including drug coverage) to protect women from high burdens for out-of-pocket medical expenses [26]. Aggravating this is the fact that women consume more medication than men [27], for example for not incurred complementary and alternative medicine [28]. Considering that women in general have to pay more out-of-pocket for medication and special equipment, as Lapsley *et al*. illustrate [29], they are more likely to rely on the aforementioned strategies to reduce costs [8]. Furthermore, gender-specific morbidities like breast cancer cause higher OOPP, since appropriate health-care plans have less generous benefits [27]. An additional aspect is women's longer life expectancy, thereby producing the greater likelihood for chronic diseases in general [4]. For financial issues, it is also relevant that women, due to their longer lifespan, have a higher probability become a widow. Those in the lowest income quartile (below $12.000) pay approximately 70% of their income in their final two years for health-care, whereas the average is around 30%, leading to higher poverty rates (17% vs. 5%). Among other reasons, OOPP in the last year of the spouses' life and the loss of the spouses' income raise the likelihood of old age poverty for widows by 56% [5]. Rice *et al*. deliver proof that if widowed women benefit from an inheritance, they are very likely to be excluded from needed low-income subsidies, because their assets exceed the limit Medicare Part D foresees, so that the assets first have to be spent out-of-pocket before the federal government steps in: 46% of those failing the asset test are widowed, and almost all are female (~93%) [6].

## Discussion

### Findings

Most of the reviewed studies describe inequalities in OOPP for medical services, in relation to diverging income proportions. The largest amount of OOPP can be assigned to prescription medications, leading to cost-induced strategies like non-adherence, implicating further health problems. This effect is further enhanced by the widespread lack of preventive measures among low-income beneficiaries, partly caused by lack of supplemental insurance. This describes women and low-educated layers of society. Considered as percentage share, women, lower social and low-income classes are far heavier affected. While these variables explain a great part of income inequalities, which finally lead to inequalities in OOPP, they are also predictors of inequalities in their own right. For example, being female and low-educated leads to being employed less or working in worse paid jobs than higher-educated people. A cycle of low income, no supplemental insurance, less paid services, worse health and thereby financially unfeasible need is set in motion. Besides inequalities associated with lower income, several studies indicate higher out-of-pocket burdens on a gender- and education-specific level. Concerning sex, gender-specific illnesses (like breast cancer or other chronic diseases that are more likely to occur due to a longer lifespan), and higher costs for medications and special equipment influence the health-care burden without being associated with income. On the education side, problems like lower awareness of a healthy lifestyle, reflecting less use of preventive measures, may constitute income-independent disadvantages. Furthermore, another effect can be observed: Neuman *et al*. [30] show that the implementation of Medicare Part D in 2006, resulting in a rising number of private insurers, led to a larger variety of available plans to choose from. This could have left low-educated beneficiaries in a confusing situation, making it hard to find adequate insurance options trying to face all possibilities and background information. As public health policy is unable to influence education's role in the short-term by providing information and transparency, possible approaches to a solution should be based on the strong correlation of income and education. Thus, appropriate measures ought to be largely coherent with those for income and burden, for example income thresholds according to beneficiaries' burden. Besides education, an enhanced preference of low-risk beneficiaries in popular plans could have been initiated by the insurers, leaving disadvantaged seniors aside. This could explain why despite all reform efforts since 2006, the number of about four million beneficiaries without drug coverage remains constant up to today. As several studies reviewed show the importance of access to prescription drug coverage for the amount of OOPP prior to the implementation of Medicare Part D in 2006 [10-12,14,17-20,22,23,26,27,31], its initiation should have settled this problem. But as studies analyzing data after 2006 reveal [8,30,32], details like the "Donut Hole" coverage gap still remain responsible for inequalities. Based on the studies reviewed, an explanation for inequalities in OOPP by these factors remains ambiguous. Also, methodically, the diversity of controlled confounders could have caused difficulties in comparing results.

### Context & theories

To give a starting point for subsequent studies examining mechanisms causing these inequalities, hereafter, the findings will shortly be put in conjunction with relevant theories. The aim is to show what other variables could cause effects on OOPP inequalities. Mojtabai *et al*. [23] demonstrated that severe health problems lead to more medication, and thereby higher OOPP, resulting in a higher probability of non-adherence, poorer health and more hospitalizations, especially for those without supplemental insurance and low income paired with high OOPP. This summary is quintessential for most of the findings in this review. The coherence between high OOPP and non-adherence to medication is observed by several authors [8,21,22], as well as the association of cost-induced strategies and worse health outcomes [19]. The intensification of this problem for low-income beneficiaries in a disadvantageous insurance situation is also evident [22]. As this finding refers to income only, Sambamoorthi *et al*. [10] put other variables in perspective by arguing that generation-comprehensive predispositions may impact actual developments. Parents' low income leads to poor education. A corresponding workplace, which results in low income (and a lack of sufficient assets), leads to a lifestyle detrimental to good health and preventive measures, which itself leads to high OOPP that one cannot afford. Reinforcing this development, inequalities intensify with age. The connection of low education, poorly paid jobs and corresponding income and insurance, is supported by several studies reviewed [8,25]. Also, coherence is found between less income and a lack of awareness of health, reflecting less adherence to medication and preventive measures [19]. The resulting effect of higher OOPP has also been demonstrated [15,22]. To put all questioned variables in perspective, several articles confirm that being female is an additional aspect worsening the situation by, for example, lower income and a longer lifespan [5,6,17,26]. So generally, Sambamoorthi *et al*.'s thesis can be confirmed. But it also becomes clear that the variables income, education and gender cannot explain the whole context independently, as other aspects throw different light on the argumentation. Ahrens [33] also identifies overall working-, living- and environmental circumstances as main influences on health status. He states that predisposing factors (age, sex, congenital diseases), medical infrastructure (quantity and quality, financial means), social status (lifestyle, income, education) and comprehensive variables (education system, environmental quality, labour conditions) as individual and systematic circumstances finally determine the means to deal with high OOPP. Therefore, it can be assumed that a concluding bottom line of coherence cannot be drawn solely on the basis of income, education and gender, as numerous other variables seem to bear an unexplored amount of influence, whose extent has to be analyzed in future studies.

### Approaches to a solution

Finally, a short windup addressing possible approaches to overcome inequalities in burden will be provided. Although beneficiaries can influence their situation by, for example, granting preventive measures a high priority, their information deficit puts them in a dependent position. Their state is defined by the physician's diagnosis, who profits from the divide in information, competence and a lack of transparency concerning the iatrogenic costs [33]. This may be a point to exercise pressure, by developing a system not paying the physician by the number of measures prescribed, but by the quality of care, reflecting his patients' health status. McCormick *et al*. [34] found that only 9.1% of US-physicians probed support the status quo, while reform proposals like adding tax credits or tax penalties (49.2%) or single payer NHI programs (41.6%) are favoured. When raising OOPP is necessary due to fiscal reasons, it should occur in relation to the patient's income. Chandra *et al*. further suggest that one regards health status as a second factor to determine the income-related limit of cost-sharing in order to protect chronically ill patients from catastrophic expenditures [31]. The focus should lie on equal burdens, not equal expenditures. By doing so, inequalities could be alleviated. Another problem to solve is "cream skimming", the insurer's selection of low-risk beneficiaries. As this issue is intensified by an increasing number of plans to choose from, the variety of plans should be reduced to a reasonable and transparent extent. Also, the government could limit the influence of private insurers on the market, so that high-income beneficiaries cannot exclude themselves from the solidarity system, leaving society socially unbalanced [30]. Additionally, the inequality problems in connection with the Donut Hole [8,18] and the Medicare Part D asset test [6] should be identified as a financial threat. If OOPP have to be imposed to stabilize the budget situation for future tasks, their implementation should be carefully thought over, with great importance attached to socially balanced arrangements.

## Limitations

Most of the studies included in the review contained a number of limitations, reflecting in a confined validity of this article, namely possible biases in inferring causal relationships from cross-sectional data, response and recall errors as a potential concern with survey data, the different approaches of data collection in reviewed surveys, and the reliability of self-reported data used in several studies. Further, comparability may be limited due to diverse confounders controlled for. Also, the impact of the implementation of Medicare Part D in 2006 and the effects of its design can not be totally clarified.

## Conclusions

As evidently shown in this systematic review, income has a significant influence on the amount of OOPP resting on the beneficiary, especially concerning the inequality of burden manifesting a profound disadvantage for low-income patients. Not only resulting in unfeasible financial difficulties, but also in worse health status, this inequity creates a vicious cycle hard to escape. Secondly, while most studies found educational and gender inequalities to be associated with income, there might also be effects induced solely by education; for example, an unhealthy lifestyle leading to higher payments for lower-educated people, or exclusively gender-induced effects, like sex-specific illnesses. Based on the considered studies, an explanation for inequalities in OOPP by these factors remains ambiguous.

## Competing interests

The authors declare that they have no competing interests.

## Authors' contributions

SC conceived the study, participated in its design and implementation, and drafted and finalized the manuscript. DH conceived the study, participated in its design and implementation, and helped to draft the manuscript. HM and TL contributed to the manuscript and with references. ER helped to draft the manuscript and commented the manuscript. HHK conceived the study, participated in its design and implementation, and helped to draft the manuscript. All authors read and approved the final draft of the manuscript.
